# Dose-dependent effects of Metformin on proliferation and odontogenic differentiation of dental pulp stem cells

**DOI:** 10.1186/s12903-026-08221-w

**Published:** 2026-04-13

**Authors:** Rania Rashad Omar Omar Taha, Nashwa El-Khazragy, Shereen Hafez Ibrahim, Nermine Hassan

**Affiliations:** 1 Department of Conservative Dentistry, Faculty of Dentistry, Suez University, Suez, Egypt; 2 Department of Clinical Pathology-Hematology and AinShams Medical Research Institute (MASRI), Faculty of Medicine, Ain Shams University, Cairo, 11566 Egypt; 3 Department of Conservative Dentistry, Faculty of Dentistry, Cairo University, Cairo, 11553 Egypt; 4 Department of Endodontics, Faculty of Dentistry, Cairo University, Cairo, 11553 Egypt

**Keywords:** Dental pulp stem cells (DPSCs), Metformin, Odontogenic differentiation, Alkaline phosphatase (ALP), Regenerative endodontics

## Abstract

**Background:**

Metformin has emerged as a potential bioactive agent in regenerative medicine; however, its optimal concentration for enhancing dental pulp stem cell (DPSC) function remains unclear. This study evaluated the dose-dependent effects of Metformin (500 µM and 1000 µM) on DPSC proliferation and odontogenic differentiation.

**Methods:**

Cell viability was assessed after 48 h, while differentiation was examined using alkaline phosphatase (ALP) activity at days 3 and 7, Alizarin Red S staining for mineralization at days 7 and 14, and gene expression analysis of *RUNX2*, *DMP1*, and *BMP2* at day 14.

**Results:**

Low-dose Metformin (500 µM) significantly increased cell viability, enhanced ALP activity, promoted mineralized matrix deposition, and upregulated odontogenic gene expression compared to control cultures. In contrast, the higher concentration (1000 µM) attenuated proliferation and differentiation markers, demonstrating a biphasic biological response.

**Conclusion:**

These findings indicate the presence of a therapeutic window in which Metformin optimally stimulates odontogenic maturation without inducing inhibitory effects. The results provide biologically grounded evidence supporting the future integration of controlled-dose Metformin into pulp capping materials and regenerative endodontic therapies, while emphasizing the necessity of precise dose optimization for clinical translation.

**Graphical abstract:**

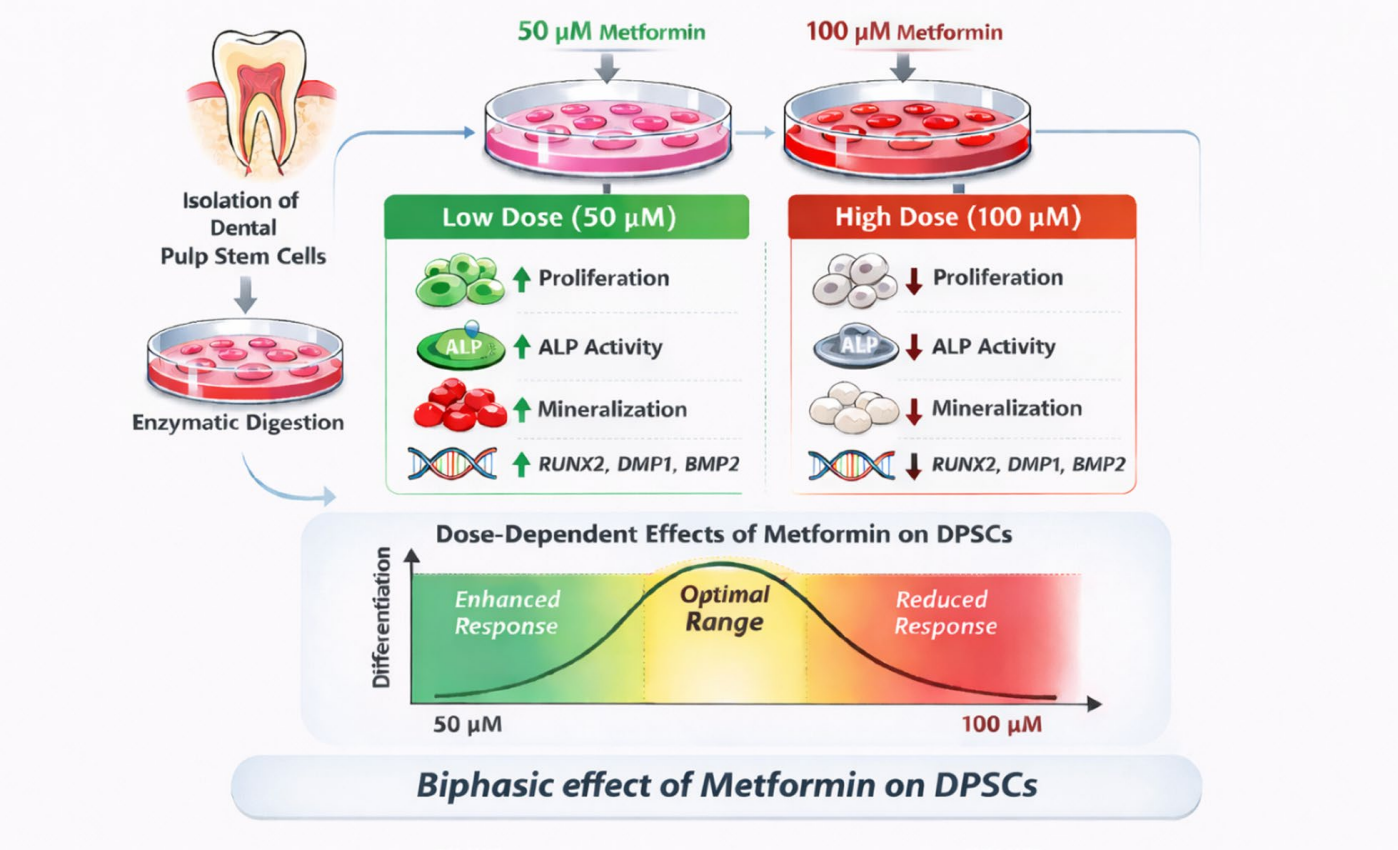

## Introduction

The preservation of dental pulp is fundamental to maintaining tooth vitality, structural integrity, and long-term oral function [1]. Dental pulp plays a critical role in dentin formation, immune defense, and sensory response, making its survival particularly important when exposed due to caries progression, operative procedures, or traumatic injury [2]. Once the pulp is compromised, inflammatory cascades and microbial invasion can rapidly lead to irreversible pulpitis or necrosis, ultimately necessitating endodontic intervention or tooth extraction [3]. Therefore, contemporary restorative dentistry increasingly emphasizes pulp preservation rather than pulp removal, aligning with minimally invasive and biologically driven treatment philosophies [4].

Vital pulp therapy (VPT) represents a cornerstone strategy aimed at maintaining pulp vitality by protecting the exposed pulp tissue and promoting its healing and regenerative capacity [4, 5]. Procedures such as direct pulp capping, indirect pulp capping, and partial pulpotomy are designed to control inflammation, prevent bacterial contamination, and stimulate reparative dentin formation [6]. The success of VPT depends largely on the biological response of dental pulp stem cells (DPSCs) [7], which possess self-renewal ability and the potential to differentiate into odontoblast-like cells, adipocytes, and neural-like cells, making them an attractive cell source for regenerative medicine and tissue engineering applications [8]. Importantly, DPSCs play a central role in the regeneration of the dentin–pulp complex due to their ability to produce mineralized extracellular matrix and contribute to reparative dentin formation following injury. Their accessibility, strong regenerative potential, and immunomodulatory properties further highlight their relevance in regenerative endodontic therapies and dental tissue engineering strategies [9]. Consequently, modern pulp therapy has shifted from passive sealing approaches toward bioactive materials capable of inducing odontogenic differentiation and pulp regeneration rather than merely acting as barriers [10].

To achieve these goals, several pulp capping agents have been developed and clinically implemented [11], including calcium hydroxide, mineral trioxide aggregate (MTA), and calcium silicate–based materials [12, 13]. Their effectiveness is commonly evaluated based on biocompatibility, antibacterial properties, sealing ability, and, most importantly, their capacity to induce odontogenic differentiation and reparative dentin formation [14]. While materials such as MTA demonstrate favorable biological outcomes, limitations including high cost, handling difficulties, discoloration potential, and prolonged setting time continue to drive the search for alternative or adjunctive bioactive agents that can enhance pulp healing at the cellular and molecular levels [15, 16].

Metformin, a widely prescribed biguanide for the management of type 2 diabetes mellitus, has recently attracted significant attention beyond its glycemic control properties [17]. Characterized by its chemical stability [18], low cost, and well-established safety profile, Metformin has demonstrated pleiotropic biological effects, including anti-inflammatory, antioxidant, and pro-regenerative actions [19, 20]. In regenerative medicine, Metformin has been shown to promote osteogenic and odontogenic differentiation of mesenchymal stem cells through modulation of cellular energy metabolism [21], positioning it as a promising bioactive molecule for dental tissue engineering applications [22, 23].

Mechanistically, Metformin primarily exerts its biological effects via activation of the AMP-activated protein kinase (AMPK) signaling pathway, a central regulator of cellular energy homeostasis [24, 25]. AMPK activation has been linked to enhanced cell survival, controlled proliferation, and upregulation of odontogenic markers such as dentin sialophosphoprotein (DSPP) and dentin matrix protein-1 (DMP-1) in dental pulp stem cells [26, 27]. Importantly, Metformin exhibits excellent biocompatibility at appropriate concentrations, with minimal cytotoxicity reported in various stem cell populations, supporting its potential use in dental regenerative applications [27].

Despite these promising attributes, the application of Metformin in pulp capping and vital pulp therapy is not without limitations [28]. Emerging evidence indicates that Metformin exerts dose-dependent effects [29, 30], where low concentrations may enhance cell proliferation and differentiation [31], while higher doses can suppress cellular activity or induce cytotoxic responses [32, 33]. This dual behavior raises critical concerns regarding optimal dosing strategies, particularly in the confined and sensitive pulp microenvironment [34]. Currently, there is no consensus on the ideal concentration range that balances regenerative stimulation without compromising pulp cell viability [35].

Notably, while several studies have explored the odontogenic potential of Metformin [36], direct comparative evaluations of different Metformin doses on both proliferation and odontogenic differentiation of dental pulp stem cells remain limited. This represents a significant gap in knowledge that hinders the rational translation of Metformin into pulp capping protocols and bioactive dental materials.

Therefore, the present study is novel in systematically comparing the effects of two distinct Metformin doses, 500 and 1000 mg, on the proliferative capacity and odontogenic differentiation potential of dental pulp stem cells. By clarifying dose-dependent cellular responses, this work aims to provide biologically grounded evidence to support the future development of Metformin-enhanced pulp capping strategies and regenerative endodontic therapies.

## Materials and methods

### Isolation, culture, and characterization of human dental pulp stem cells

Human dental pulp stem cells (h-DPSCs) were isolated from freshly extracted, caries-free human third molars/premolars obtained under approved ethical clearance and written informed consent. Immediately after extraction, teeth were rinsed thoroughly with sterile DPBS without Ca²⁺/Mg²⁺ (Gibco; Thermo Fisher Scientific), disinfected externally, and sectioned under aseptic conditions to expose the pulp chamber. Pulp tissues were gently extirpated, minced into 1 mm³ fragments, and enzymatically digested using Collagenase Type I (Gibco; Thermo Fisher Scientific) in combination with Dispase II (Gibco; Thermo Fisher Scientific) to obtain a single-cell suspension. The digest was neutralized with complete culture medium and centrifuged; the cell pellet was resuspended and seeded in tissue-culture-treated flasks. Cells were cultured at 37 °C in a humidified 5% CO₂ incubator using α-MEM (MEM α, nucleosides) (Gibco; Thermo Fisher Scientific) supplemented with Fetal Bovine Serum (FBS), qualified (Gibco; Thermo Fisher Scientific), and Penicillin–Streptomycin (10,000 U/mL) (Gibco; Thermo Fisher Scientific). Medium was replaced every 3 days, and adherent fibroblast-like colonies were expanded. Upon reaching 70% confluence, cells were detached using TrypLE™ Express Enzyme (1X), no phenol red (Gibco; Thermo Fisher Scientific), and passaged for downstream assays; cells between early passages (P2–P4) were used for characterization to minimize culture-induced drift. For immunophenotypic characterization, harvested cells were washed in DPBS and stained according to the manufacturer’s recommendations with fluorochrome-conjugated anti-human antibodies against CD105 (MA1-19594) FITC, CD90-FITC Monoclonal Antibody (11-0903-82), and CD45-FITC (35-0451-82) FITC monoclonal antibodies (***eBioscience™***,*** Thermofisher Scientific***,*** USA***). Flow cytometric acquisition and analysis were performed on the NAVIOS EX 10-color flow cytometer (Beckman Coulter), and data were analyzed with Navios software, and h-DPSCs were defined by the expected mesenchymal profile (CD105⁺/CD90⁺/CD45⁻) [37].

### Preparation of metformin solution

Metformin hydrochloride (molecular weight 165.63 g/mol; *Sigma-Aldrich*,* St. Louis*,* MO*,* USA*) was used for all experiments. A sterile stock solution (100 mM) was prepared by dissolving 165.6 mg of Metformin hydrochloride in sterile cell culture-grade distilled water to a final volume of 10 mL. The solution was mixed until complete dissolution was achieved and subsequently sterilized by filtration through a 0.22 μm syringe filter under aseptic conditions. The prepared stock solution was aliquoted and stored at − 20 °C until further use to avoid repeated freeze–thaw cycles.

Working concentrations of 500 µM and 1000 µM were prepared freshly by dilution of the 100 mM stock solution in osteogenic differentiation medium (ODM) using the dilution equation (C₁V₁ = C₂V₂). For the preparation of 500 µM Metformin, 50 µL of the 100 mM stock solution was added to 10 mL of ODM. For the preparation of 1000 µM Metformin, 100 µL of the 100 mM stock solution was added to 10 mL of ODM. The supplemented media were gently mixed to ensure homogeneity and were used immediately for cell treatment.

### Metformin treatment and cell proliferation assessment

Dental pulp stem cells (DPSCs) were treated with Metformin to investigate its dose-dependent effects on cell proliferation. Metformin hydrochloride was obtained from Thermo Fisher Scientific (Thermo Scientific™ grade) and freshly prepared in sterile culture medium prior to each experiment. Following cell attachment and stabilization after 24 h, DPSCs were allocated into three experimental groups: a control group cultured in complete growth medium without Metformin, and two treatment groups cultured in medium supplemented with Metformin 500 µM and 1000 µM, respectively. The Metformin stock solution was prepared by dissolving Metformin hydrochloride powder in sterile water and adjusting the concentration to achieve the desired final working concentrations in the culture medium. Cells were maintained at 37 °C in a humidified atmosphere containing 5% CO₂.

Cell proliferation was assessed after 48 h of treatment using the Vybrant^®^ MTT Cell Proliferation Assay Kit (Thermo Fisher Scientific), according to the manufacturer’s instructions. Briefly, MTT reagent was added to each well and incubated to allow mitochondrial dehydrogenases of viable cells to convert the tetrazolium salt into insoluble formazan crystals. Following incubation, the resulting formazan was solubilized, and absorbance was measured spectrophotometrically at a 570 nm wavelength using a microplate reader. Proliferative activity of Metformin-treated DPSCs was expressed relative to untreated control cells, enabling quantitative comparison of dose-dependent effects on cell viability and metabolic activity [38].

### Assessment of odontogenic differentiation of DPSCs

The odontogenic differentiation of dental pulp stem cells (DPSCs) was evaluated by assessing alkaline phosphatase (ALP) activity, mineralization, and the expression of odontogenic-associated genes at multiple time points. For ALP activity, cells were cultured in odontogenic differentiation medium and treated with Metformin for 3 days and 7 days. The ALP activity was assessed using the ALP Assay Kit (Sigma-Aldrich; Germany), following the manufacturer’s instructions. Absorbance was measured spectrophotometrically at a wavelength of 405 nm, with activity expressed relative to total protein content [39]. To assess mineralization, Alizarin Red S staining (Sigma-Aldrich) was performed at days 7 and 14. Briefly, cultures were fixed, stained with Alizarin Red for 30 min, and then washed to remove unbound dye. Mineralized nodules were visualized and quantified by measuring the absorbance of the extracted stain at 540 nm [40].

To evaluate gene expression, total RNA was extracted from DPSCs at day 14 using the Qiagen RNeasy Mini Kit (Qiagen, Germany). RNA quality and concentration were assessed spectrophotometrically, and cDNA was synthesized using the Qiagen QuantiTect Reverse Transcription Kit. Quantitative PCR was performed using the Qiagen QuantiTect SYBR Green PCR Kit and the Quantitect primer assay (cat no: 249900) for Hs_*RUNX2*_1_SG, assay ID: QT00020517, Hs_*DMP1*_1_SG, assay ID: QT00022078, and Hs_*BMP*_vc.2_SG, assay ID: QT00024535, to assess the expression of odontogenic-related genes, with the β-actin Hs_*ACTB*, ID: QT000954231, used as the housekeeper gene. PCR amplification conditions were optimized according to the manufacturer’s protocols, and the relative gene expression levels were calculated using the ΔΔCt method [41].

### Statistical methods

Statistical analyses were conducted using GraphPad Prism version 9 (GraphPad Software, San Diego, CA, USA). Before performing any analysis, data were checked for normality and homogeneity of variance. A one-way analysis of variance (ANOVA) was used to determine overall differences between multiple groups, with Tukey’s post-hoc test applied for pairwise comparisons. Results are presented as the mean ± standard deviation, and a p-value < 0.05 was considered statistically significant. Graphs were created using Prism’s built-in visualization tools to maintain consistent formatting and ensure accurate data representation.

## Results

### Characterization and morphological validation of isolated DPSCs

**Fig. 1:**
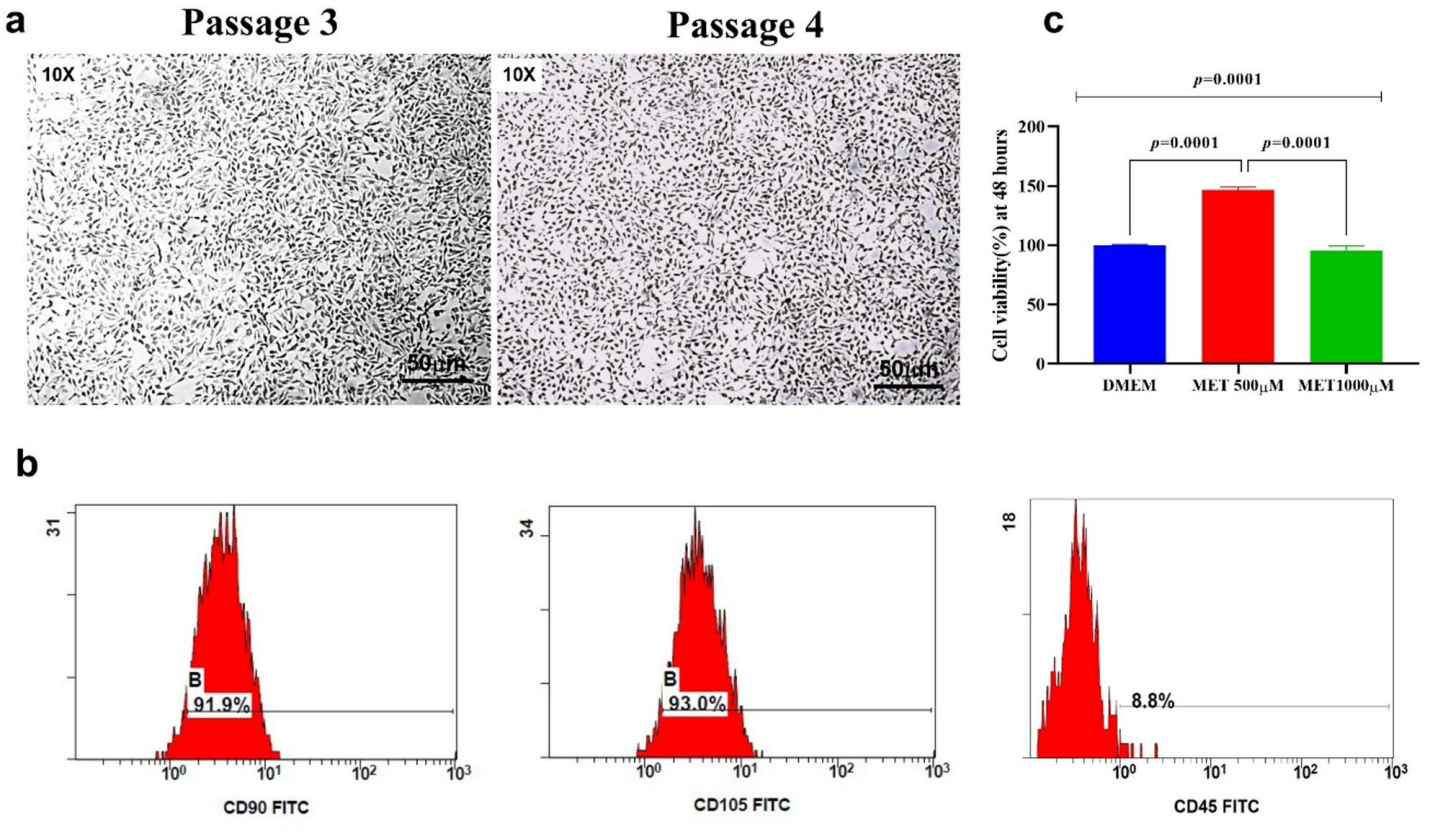
Characterization and Effects of Biomaterials on DPSCs:** a** h-DPSCs at P3 and P4 showing typical well-differentiated morphology with flattened spindle-shaped cells and hyperchromatic nuclei (20× magnification, scale bar: 20 μm). **b** Flow cytometry dot plots for DPSCs stained with CD105-FITC, CD90-FITC and CD45,-CD45-FITC showing high CD90 expression (91.9%), CD105 (93.0%), and low CD45 expression (8.8%), confirming the purity of DPSCs. **c** Bar graph comparing DPSC viability after 48 h of treatment with low dose (500µM), and high dose (1000 µM) of Metformin, showing significantly higher viability in the low-dose group. Data are presented as mean ± SD, with statistical significance determined by ANOVA and Tukey’s post-hoc test (*p* < 0.05)

Microscopic examination of cultured human dental pulp stem cells (h-DPSCs) at passage 3 (P3) and passage 4 (P4) demonstrated a stable and homogeneous mesenchymal phenotype, consistent with successful isolation and expansion. As shown in Fig. 1a, cells exhibited the classical fibroblast-like morphology characterized by flattened, spindle-shaped architecture and elongated cytoplasmic processes. The nuclei appeared hyperchromatic and centrally located, reflecting active proliferative capacity. At P3, cells displayed well-defined spindle contours with moderate confluency, indicating early stabilization after primary culture. By P4, the culture exhibited increased cellular density with maintained morphological uniformity, suggesting preservation of stemness and absence of spontaneous differentiation. Importantly, no morphological heterogeneity, epithelial-like transformation, or senescence-associated enlargement was observed, confirming phenotypic stability across passages.

To validate the mesenchymal origin and purity of the isolated cell population, immunophenotypic characterization was performed using flow cytometry (Fig. 1b). The analyzed cells demonstrated strong expression of CD90 and CD105, two well-established positive markers of mesenchymal stem cells (MSCs), confirming their stromal lineage identity. Conversely, minimal expression of CD45, a hematopoietic lineage marker, was detected, effectively excluding contamination with blood-derived or immune cells. The clear separation between positive and negative populations in the dot plots further supports the specificity of staining and the homogeneity of the cultured cells.

Collectively, the preserved spindle-shaped morphology across passages and the characteristic MSC immunophenotypic profile (CD90⁺/CD105⁺/CD45⁻) confirm successful isolation, expansion, and purity of DPSCs suitable for downstream experimental applications.

### Dose-dependent effect of Metformin on the biocompatibility of DPSCs

The descriptive statistics presented in Table 1 and illustrated graphically in Fig. 1c demonstrate a clear dose-dependent effect of Metformin on DPSC viability after 48 h of exposure. Cells cultured in DMEM alone maintained baseline viability, serving as the physiological control condition. In contrast, treatment with low-dose Metformin (500 µM) resulted in a marked enhancement of cell viability compared to the control group (*p* = 0.0001), indicating a stimulatory or proliferative-promoting effect at this concentration. The magnitude of increase was statistically significant, confirming that the observed enhancement was not due to random variation. Conversely, exposure to high-dose Metformin (1000 µM) led to a significant reduction in cell viability compared not only to the low-dose group but also relative to the DMEM control.

**Table 1:** Descriptive statistics of cell viability percentage in the studied groups after 48 h

Group	mean ± SD	Range	*p*-value
DMEM	100 ± 0.75	99.3–101	
Metformin 500 mg	146 ± 2.64	143–148	0.0001 ^a/c^
Metformin 1000 mg	95.4 ± 3.97	90.9–98.1	0.0001 ^b^

*F *Statistical value of ANOVA test, *ANOVA *Analysis of variances test, *DPSCs *Dental pulp-derived mesenchymal stem cells, *DMEM *Dulbecco’s Modified Eagle Medium (DMEM)

^a^statistical significance compared to the DPSCs “untreated cells” (*p* < 0.05)

^b^statistical significance compared to the low dose of MET (500 mg) (*p* < 0.05)

^c^statistical significance compared to the high dose of MET (1000 mg) (*p* < 0.05)

### Dose-dependent effect of metformin on ALP activity during odontogenic differentiation of DPSCs

**Fig. 2:**
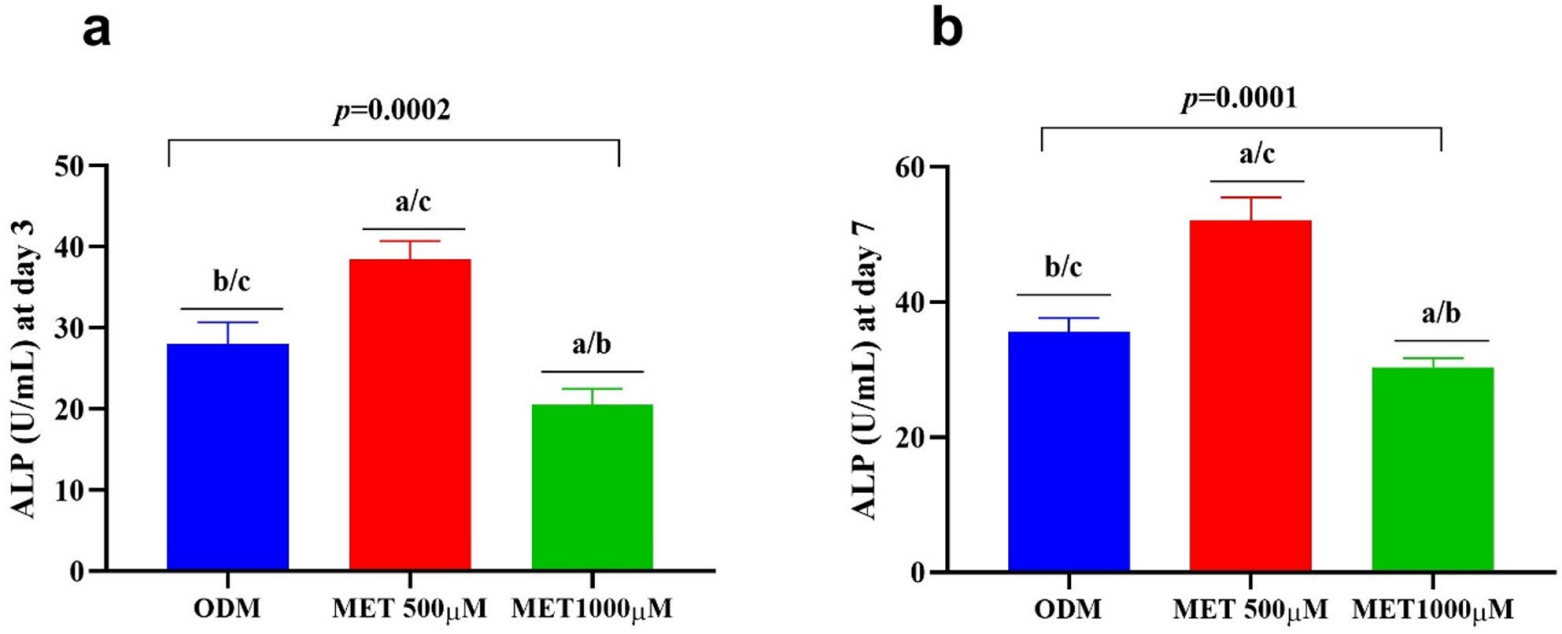
Osteogenic differentiation of DPSCs cultured with ODM, a low dose of Metformin (500 µM), and a high dose of Metformin (1000 µM) for 3 and 7 days. Bar graphs (**a, b**) display ALP activity (U/mL) after 3 days and 7 days of exposure; respectively, showing progressively increasing osteogenic responses from Metformin with two different doses, compared to cells cultured in ODM. Error bars represent standard deviations. Statistical significance was assessed using one-way ANOVA followed by Tukey’s post-hoc test (^a^: vs. ODM; ^b^: vs. low dose of Metformin; ^c^: vs. high dose of Metformin. Abbreviations: DPSCs: Dental pulp-derived mesenchymal stem cells, ODM: Osteogenic differentiation medium, MET: Metformin, ALP: Alkaline phosphatase

The data presented in Table 2 and illustrated in Fig. 2 demonstrate a clear concentration and time-dependent modulation of alkaline phosphatase (ALP) activity in DPSCs undergoing odontogenic differentiation. At day 3 (Fig. 2a), cells cultured in osteogenic differentiation medium (ODM) exhibited baseline induction of ALP activity, confirming initiation of early odontogenic differentiation. However, supplementation with low-dose Metformin (500 µM) significantly enhanced ALP activity compared to ODM alone (*p* = 0.0001), indicating an accelerated early differentiation response. In contrast, exposure to high-dose Metformin (1000 µM) resulted in a significant reduction in ALP activity relative to both ODM and the low-dose group, suggesting an inhibitory effect at higher concentrations during the early differentiation phase.

By day 7 (Fig. 2b), ALP activity increased across all groups, reflecting the expected progression of differentiation over time. Nevertheless, the magnitude of response differed markedly among treatments. The low-dose Metformin group demonstrated the highest ALP activity, significantly exceeding both the ODM and high-dose groups, indicating a sustained and potentiated odontogenic response. Cells cultured in ODM alone showed moderate ALP upregulation consistent with normal differentiation kinetics. Conversely, the high-dose Metformin group exhibited significantly lower ALP activity compared to the low-dose group and remained inferior to ODM at this later time point, reinforcing the suppressive effect of excessive Metformin concentration on differentiation capacity. Statistical analysis using one-way ANOVA followed by Tukey’s post-hoc testing confirmed significant differences among groups at both time points.

**Table 2:** Dose-dependent effect of metformin on odontogenic differentiation of DPSCs

Variable	ODM	Metformin 500 µM	Metformin 1000 µM	*p*-value
ALP (U/mL) at day 3	28.0 ± 2.67^b/c^	38.5 ± 2.26^a/c^	20.5 ± 1.9^a/b^	0.0002
ALP (U/mL) at day 7	35.6 ± 2.08^b^	52.1 ± 3.4^c^	30.3 ± 1.34^a/b^	0.0001
ARS (µM) at day 7	1.74 ± 0.11^b/c^	3.22 ± 0.15^a/c^	2.05 ± 0.12^a/b^	0.0001
ARS (µM) at day 14	5.01 ± 0.45^b/c^	6.81 ± 0.53^a/c^	2.75 ± 0.3^a/b^	0.0001

### Effect of metformin concentration on matrix mineralization of DPSCs assessed by alizarin Red S staining

**Fig. 3:**
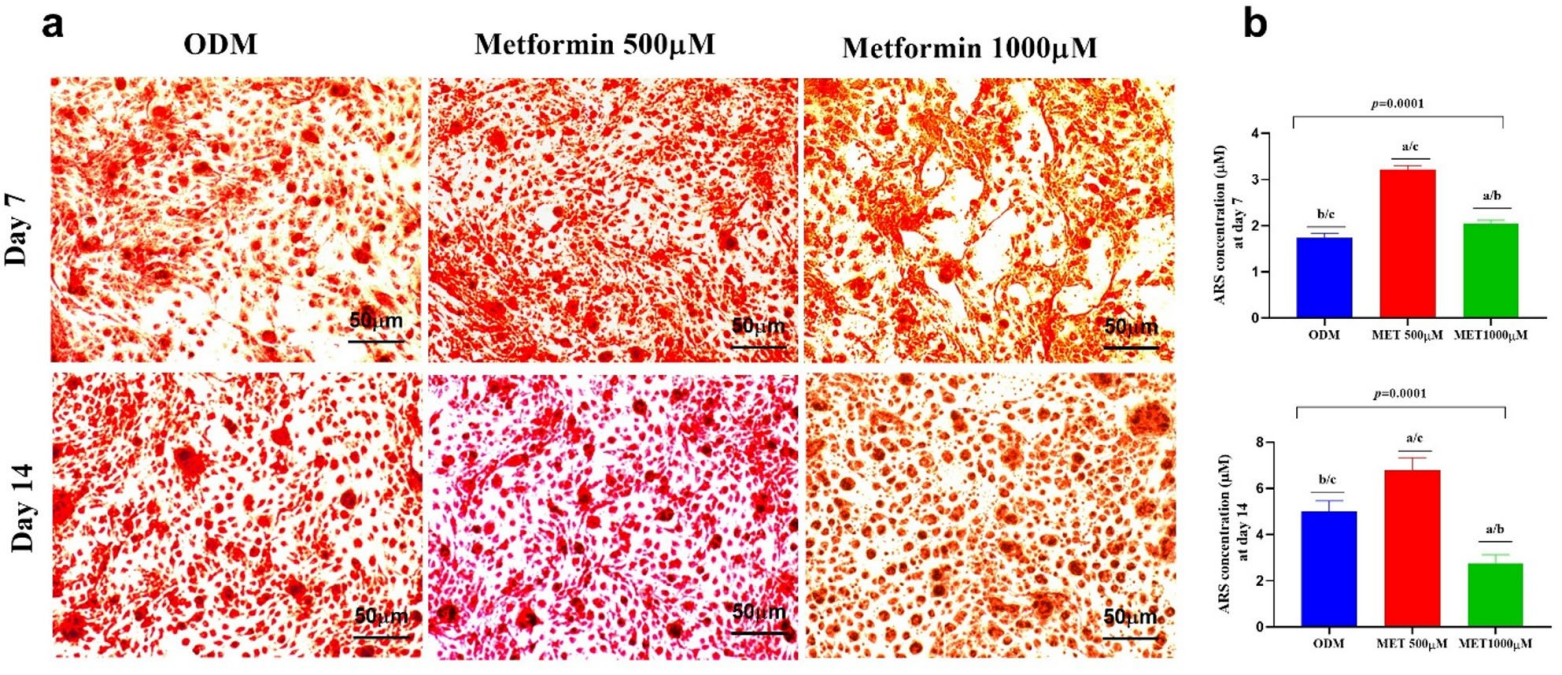
Alizarin Red S staining and ARS quantification of mineralized matrix in DPSCs cultured under different conditions for 7 and 14 days. **a** Representative images show calcium deposition in DPSCs cultured in osteogenic differentiation medium (ODM) alone (control), ODM supplemented with a low dose of Metformin (500 µM), or a high dose of Metformin (1000 µM). The magnification is 20×, and the scale bar is 50 μm. Images were captured using the LABOMED Trinocular inverted phase contrast microscope model TCM400 and Atlas 16MP CMOS USB Camera with PixelPro 3.0 software (LABOMED, USA). **b** Bar graphs show ARS concentrations representing mineral deposition at days 7 and 14. Error bars represent standard deviations. Statistical significance was assessed using one-way ANOVA followed by Tukey’s post-hoc test (a: vs. ODM; b: vs. low dose of Metformin; c: vs. high dose of Metformin. Abbreviations: DPSCs: Dental pulp-derived mesenchymal stem cells, ODM: Osteogenic differentiation medium, MET: Metformin, ARS: Alizarin red stain

The quantitative data presented in Table 2 and the representative micrographs (Fig. 3a) and bar charts in **(**Fig. 3b**)** demonstrate a pronounced dose- and time-dependent modulation of mineralized matrix formation in DPSCs. At day 7, cells cultured in osteogenic differentiation medium (ODM) exhibited initial mineral deposition, reflecting early-stage extracellular matrix calcification. Supplementation with low-dose Metformin (500 µM) markedly enhanced Alizarin Red S (ARS) staining intensity and quantification compared to ODM alone, indicating accelerated mineralization. In contrast, the high-dose Metformin group (1000 µM) showed only a modest increase relative to ODM and significantly lower mineral deposition than the low-dose group, suggesting partial suppression of calcific maturation at this concentration.

By day 14, mineralization increased substantially in all groups, consistent with progressive odontogenic differentiation over time. However, intergroup differences became more pronounced. The low-dose Metformin group demonstrated the highest ARS quantification and densest calcium nodules microscopically, confirming a potentiated mineralization response. ODM alone showed moderate but clearly established mineralized matrix formation, representing physiological differentiation progression. Strikingly, the high-dose Metformin group exhibited significantly reduced ARS deposition compared to both ODM and low-dose groups, indicating that prolonged exposure to elevated Metformin concentrations impairs late-stage mineral maturation. Statistical analysis confirmed highly significant differences among groups at both time points.

### Dose-dependent modulation of odontogenic gene expression in DPSCs After 14 days of metformin treatment

**Fig. 4:**
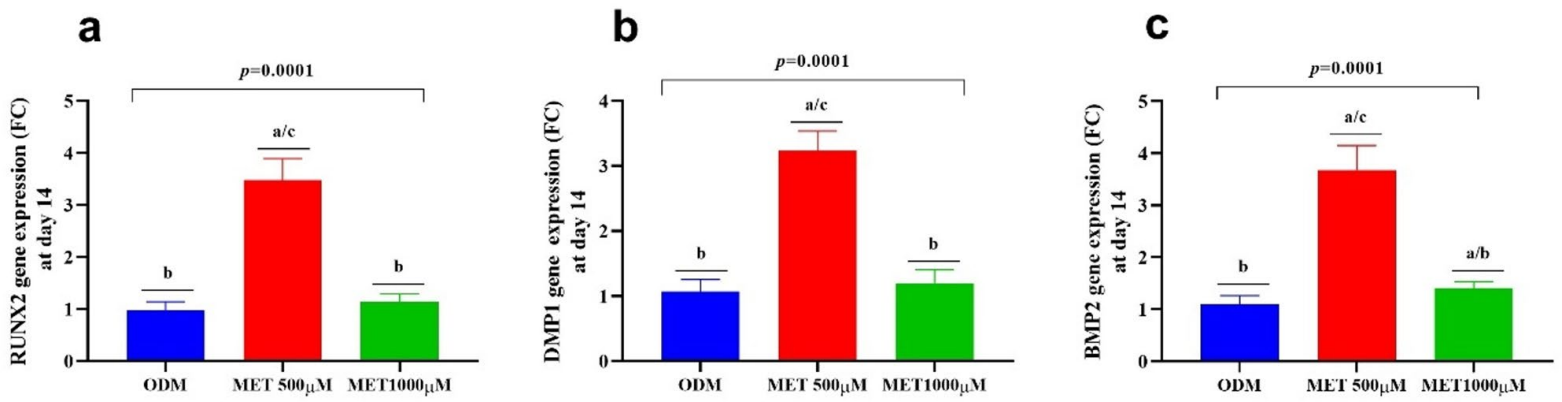
Odontogenic gene expression of DPSCs after 14 days of culture with ODM, collagen, MTA, and collagen + MTA. Bar graphs illustrate the fold-expression profiles of *RUNX2* (**a**), *DMP1* (**b)**, and *BMP2* (**c**). The figure demonstrates a graded increase in odontogenic gene activation from ODM to a low dose of Metformin, producing the strongest overall transcriptional response. Statistical significance was determined using one-way ANOVA followed by Tukey’s post-hoc test (a: vs. ODM; b: vs. low dose of Metformin (500 µM); c: vs. high dose of Metformin (1000 µM)). Abbreviations: ODM = osteogenic differentiation medium; DPSCs: Dental pulp-derived mesenchymal stem cells, ODM: Osteogenic differentiation medium, *RUNX2*: runt-related transcription factor 2, *DMP1*: dentin matrix acidic phosphoprotein 2, *BMP2*: bone morphogenetic protein 1, FC: fold change

The quantitative gene expression analysis presented in Table 3 and illustrated in Fig. 4 reveals a significant concentration-dependent effect of Metformin on odontogenic transcriptional activation in DPSCs after 14 days of culture. Cells maintained in osteogenic differentiation medium (ODM) exhibited baseline expression of *RUNX2*, *DMP1*, and *BMP2*, reflecting standard differentiation signaling. However, supplementation with low-dose Metformin (500 µM) resulted in a marked upregulation of all three odontogenic markers, demonstrating a robust enhancement of transcriptional activity compared to ODM alone. This increase was statistically significant and consistent across early (*RUNX2*)(Fig. 4a), matrix-related (*DMP1*) (Fig. 4b), and morphogenetic (*BMP2*)(Fig. 4c) genes, indicating coordinated activation of odontogenic differentiation pathways.

In contrast, exposure to high-dose Metformin (1000 µM) produced only minimal elevation in gene expression relative to ODM and was significantly lower than the low-dose group. The transcriptional levels in the high-dose group remained close to baseline differentiation values, suggesting attenuation of stimulatory signaling at higher concentrations. Statistical analysis using one-way ANOVA followed by Tukey’s post-hoc test confirmed highly significant intergroup differences for all genes examined (*p* = 0.0001).

**Table 3:** Gene expression in DPSCs at day 14 of exposure to ODM and low and high doses of Metformin

RUNX2 (FC)	ODM	Metformin 500 µM	Metformin 1000 µM	*p*-value
	0.97 ± 0.16^b^	3.47 ± 0.42 ^a/c^	1.13 ± 0.16^/b^	0.0001
*DMP1* (FC)	1.07 ± 0.18^b^	3.23 ± 0.32^a/c^	1.20 ± 0.21^b^	0.0001
*BMP2* (FC)	1.10 ± 0.18^b^	3.68 ± 0.48^a/c^	1.14 ± 0.13^a/b^	0.0001

Mean ± SD values of ALP activity at day 3 and day 7 are presented for all experimental groups. One-way ANOVA showed significant differences across groups at both time points (*p* = 0.0001)

Superscript letters denote statistically significant pairwise comparisons versus ODM (a), low dose of Metformin (b), and high dose of Metformin (c)

*Abbreviations*: *ODM *Osteogenic differentiation medium, *DPSCs *Dental pulp-derived mesenchymal stem cells, *ODM *Osteogenic differentiation medium, *RUNX2 *Runt-related transcription factor 2, *DMP1* Dentin matrix acidic phosphoprotein 2, *BMP2* Bone morphogenetic protein 1, *FC *Fold change

## Discussion

The present study was designed to clarify the dose-dependent effects of Metformin on the proliferative behavior and odontogenic differentiation potential of DPSCs, with the ultimate goal of identifying a biologically rational concentration that could support future regenerative endodontic applications. The findings consistently demonstrated a biphasic response pattern across viability, enzymatic differentiation markers, mineralization capacity, and odontogenic gene expression. Collectively, the results highlight that Metformin at 500 µM enhances DPSC proliferation and differentiation, whereas 1000 µM exerts attenuating or inhibitory effects, thereby defining a therapeutic window for regenerative strategies. Importantly, cytotoxicity should not be interpreted solely as reduced cell viability but as a broader biological response that also affects cellular function and differentiation capacity. Therefore, cytocompatibility of dental pulp stem cells should be evaluated together with regenerative indicators such as ALP activity, mineralization, and odontogenic gene expression [42].

Regarding proliferative capacity, treatment with low-dose Metformin (500 µM) significantly enhanced cell viability compared to control cultures maintained in DMEM. This proliferative-promoting effect suggests that Metformin at this concentration may stimulate metabolic pathways that favor stem cell survival and expansion [43]. These findings align with previous reports demonstrating that Metformin can activate *AMP-activated protein kinase* (*AMPK*), thereby improving cellular energy balance and promoting stem cell survival under controlled conditions [44, 45]. Several studies have shown that low-dose Metformin enhances mesenchymal stem cell proliferation and protects against oxidative stress-induced apoptosis, supporting our observation of improved viability [46]. In contrast, exposure to high-dose Metformin (1000 µM) resulted in a significant reduction in viability relative to both the control and low-dose groups. Although the decrease did not indicate overt cytotoxicity, it suggests growth-inhibitory effects at high concentrations. Similar dose-dependent inhibitory effects have been reported in other stem cell populations [45], where excessive *AMPK* activation or mitochondrial interference by higher Metformin concentrations impaired cellular metabolism [47]. Thus, the observed biphasic proliferative response is consistent with the concept that Metformin exerts beneficial metabolic modulation at low doses but may induce cellular stress when administered at higher levels [48].

The differentiation findings further reinforced this concentration-dependent behavior. Low-dose Metformin significantly enhanced ALP activity at both early and later time points, indicating stimulation of early odontogenic differentiation. This observation is consistent with studies showing that Metformin promotes osteogenic differentiation of mesenchymal stem cells through *AMPK*-mediated upregulation of *RUNX2* signaling pathways [45, 49, 50]. Conversely, the high-dose group exhibited attenuated ALP activity compared to the low-dose group, suggesting suppression of differentiation signaling. Similar inhibitory trends at higher concentrations have been reported in osteoblast-like cells, where excessive Metformin exposure disrupted differentiation-related gene networks [51, 52].

Matrix mineralization, assessed by Alizarin Red S staining, followed the same biphasic trend. Low-dose Metformin markedly increased mineral deposition at both day 7 and day 14, indicating enhanced maturation and extracellular matrix calcification. These results corroborate previous in vitro studies demonstrating that Metformin stimulates mineralized nodule formation and enhances osteo/odontogenic maturation via activation of AMPK and downstream bone morphogenetic signaling pathways [45, 53]. In contrast, the high-dose group displayed reduced mineralization relative to both the ODM and low-dose groups, suggesting that excessive Metformin may impair late-stage matrix maturation. This attenuation at higher concentrations has been similarly described in bone regeneration studies where supraphysiologic Metformin levels interfered with mitochondrial respiration and reduced extracellular matrix production [52, 54].

At the molecular level, low-dose Metformin significantly upregulated key odontogenic genes, including *RUNX2*, *DMP1* (likely referring to *DMP1*), and *BMP2*, confirming transcriptional activation of differentiation pathways. These findings align with prior research indicating that Metformin enhances *RUNX2* expression and promotes osteogenic gene cascades in stem cells [49]. Specifically, recent studies have shown that Metformin-derived materials can promote the expression of DMP1 and other odontoblastic markers in human DPSCs [31]. The pronounced upregulation observed at 500 µM suggests optimal activation of signaling pathways governing matrix formation and mineralization. Conversely, the high-dose group demonstrated only modest gene expression changes, reinforcing the inhibitory shift observed in functional assays. Such dose-dependent transcriptional modulation further supports the existence of a biologically optimal concentration range [51, 55].

Overall, the consistent biphasic pattern observed across proliferation, enzymatic activity, mineralization, and gene expression strongly indicates that Metformin exerts concentration-dependent regulatory effects on DPSCs [56]. The 500 µM concentration appears to represent an optimal therapeutic range that enhances both proliferative and odontogenic capacities without inducing metabolic stress [57]. This biphasic response to metformin may reflect how formulation-related factors, including resin modification, ion release kinetics, bioactive concentrations and polymerizable additives, can influence cellular behavior and mineralization potential in dental pulp stem cells, rather than solely from the general classification of the biomaterial [58]. These findings provide biologically grounded evidence supporting the potential integration of controlled-dose Metformin into pulp capping materials and regenerative endodontic strategies, while emphasizing the necessity of precise dose optimization to avoid counterproductive inhibitory effects [59, 60].

Despite the promising findings, several limitations should be acknowledged. First, the study was conducted entirely in vitro, which does not fully replicate the complex biological microenvironment of the dental pulp in vivo, where vascular supply, immune responses, mechanical stress, and extracellular matrix interactions can significantly influence stem cell behavior. Second, only two Metformin concentrations were evaluated, which limits the ability to precisely define the full therapeutic dose-response curve or identify intermediate concentrations that might further refine the optimal range. Third, the assessment period was relatively short-term, focusing primarily on early and intermediate differentiation markers without evaluating long-term functional dentin formation or in vivo regenerative outcomes. Additionally, mechanistic pathways such as direct confirmation of AMPK activation or downstream signaling cascades were not investigated, limiting deeper molecular interpretation of the biphasic response. Finally, donor variability of DPSCs was not extensively analyzed, which may influence translational reproducibility. Therefore, future studies incorporating broader dose ranges, mechanistic pathway analysis, animal models, and long-term functional assessments are necessary to validate the clinical applicability of Metformin-enhanced regenerative endodontic strategies.

## Conclusion and future perspectives

Within the limitations of this study, Metformin demonstrated a clear dose-dependent effect on the proliferative capacity and odontogenic differentiation of dental pulp stem cells. The 500 µM concentration consistently enhanced cell viability, ALP activity, mineralized matrix formation, and odontogenic gene expression, indicating an optimal stimulatory range that supports both early and late differentiation events. In contrast, the 1000 µM concentration attenuated proliferation and differentiation responses, confirming a biphasic biological effect and emphasizing the importance of dose optimization. Collectively, these findings support the concept of a defined therapeutic window in which Metformin can enhance regenerative potential without inducing inhibitory cellular stress.

From a translational perspective, controlled low-dose Metformin may represent a promising adjunct in pulp capping materials and regenerative endodontic protocols aimed at enhancing dentin–pulp complex repair. Future investigations should focus on detailed mechanistic pathway analysis (particularly AMPK-mediated signaling), evaluation of intermediate dose ranges, long-term dentin formation studies, and validation in animal models and clinical settings. Precise pharmacological tuning will be essential to move from in vitro promise to predictable clinical regeneration.

## Data Availability

The data supporting this study’s findings are available from the corresponding author upon reasonable request.

## Abbreviations

DPSC: Dental Pulp Stem Cell
ALP: Alkaline Phosphatase
VPT: Vital Pulp Therapy
MTA: Mineral Trioxide Aggregate
AMPK: Amp-Activated Protein Kinase
DSPP: Dentin Sialophosphoprotein
DMP-1: Dentin Matrix Protein-1
BMP2: Bone Morphogenic protein 2
FBS: Fetal Bovine Serum
ODM: Osteogenic Differentiation Medium
MSC: Mesenchymal Stem Cell
ARS: Alizarin Red S
